# A Single Cell Level Based Method for Copy Number Variation Analysis by Low Coverage Massively Parallel Sequencing

**DOI:** 10.1371/journal.pone.0054236

**Published:** 2013-01-23

**Authors:** Chunlei Zhang, Chunsheng Zhang, Shengpei Chen, Xuyang Yin, Xiaoyu Pan, Ge Lin, Yueqiu Tan, Ke Tan, Zhengfeng Xu, Ping Hu, Xuchao Li, Fang Chen, Xun Xu, Yingrui Li, Xiuqing Zhang, Hui Jiang, Wei Wang

**Affiliations:** 1 Science and Technology, BGI-Shenzhen, Shenzhen, China; 2 Institute of Reproductive and Stem Cell Engineering, Central South University, Changsha, China; 3 Center of Prenatal Diagnosis, Nanjing Maternal and Child Health Hospital, Nanjing Medical University, Nanjing, China; 4 Shenzhen Municipal Key Laboratory of Birth Defects Screening and Engineering, BGI-Shenzhen, Shenzhen, China; 5 Guangdong Provincial Key Laboratory of Human Diseases Genome, BGI-Shenzhen, Guangdong, China; 6 Institute of Reproductive and Stem Cell Engineering, Central South University, Changsha, China; 7 Key laboratory of Stem Cell and Reproductive Engineering, Ministry of Health, Changsha, China; 8 State Key Laboratory of Bioelectronics, School of Biological Science and Medical Engineering, Southeast University, Nanjing, China; 9 School of Bioscience and Bioengineering, South China University of Technology, Guangzhou, China; 10 Department of Biology, University of Copenhagen, Copenhagen, Denmark; Emory University School Of Medicine, United States of America

## Abstract

Copy number variations (CNVs), a common genomic mutation associated with various diseases, are important in research and clinical applications. Whole genome amplification (WGA) and massively parallel sequencing have been applied to single cell CNVs analysis, which provides new insight for the fields of biology and medicine. However, the WGA-induced bias significantly limits sensitivity and specificity for CNVs detection. Addressing these limitations, we developed a practical bioinformatic methodology for CNVs detection at the single cell level using low coverage massively parallel sequencing. This method consists of GC correction for WGA-induced bias removal, binary segmentation algorithm for locating CNVs breakpoints, and dynamic threshold determination for final signals filtering. Afterwards, we evaluated our method with seven test samples using low coverage sequencing (4∼9.5%). Four single-cell samples from peripheral blood, whose karyotypes were confirmed by whole genome sequencing analysis, were acquired. Three other test samples derived from blastocysts whose karyotypes were confirmed by SNP-array analysis were also recruited. The detection results for CNVs of larger than 1 Mb were highly consistent with confirmed results reaching 99.63% sensitivity and 97.71% specificity at base-pair level. Our study demonstrates the potential to overcome WGA-bias and to detect CNVs (>1 Mb) at the single cell level through low coverage massively parallel sequencing. It highlights the potential for CNVs research on single cells or limited DNA samples and may prove as a promising tool for research and clinical applications, such as pre-implantation genetic diagnosis/screening, fetal nucleated red blood cells research and cancer heterogeneity analysis.

## Introduction

Copy number variations (CNVs) are known to be associated with various diseases, such as 22q11.2 deletion/duplication syndrome [1], [2], [3], Cri-du-Chat (5p deletion) [4] and even cancers [5]. Rather than cell-population research, single cell analysis provides insights into research of disease aetiology and diagnosis. It is especially useful for cancer heterogeneity research, since it has been shown to detect a single-nucleotide mutation that could result in a kidney tumor [6]. It is also useful for conducting evolution research since it has proven that CNVs plays an important role on cell evolution [7], [8]. In addition, for the clinical purposes, pre-implantation genetic diagnosis/screening (PGD/PGS) was used for disease scanning since 1990s [9], [10], [11]. This application allows CNVs analysis of single cell isolated from polar body, blastomere or blastocyst. Single-cell analysis may also open up new opportunities for noninvasive prenatal genetic diagnosis by only needing a single fetal nucleated red blood cell (NRBC) [12], [13].

Whole genome amplification (WGA) and array comparative genomic hybridization (aCGH) technology have been widely used in CNVs analysis of single cells [14], [15]. aCGH technology is based on the differential labels of test sample and reference DNA with fluorophores. These samples are then hybridized to array containing oligonucleotide probes and subsequently analyzed for fluorometric signal ratios, which allows for the calling of the copy number profile of discrete genomic intervals. However, there are several limitations of WGA-based aCGH. WGA-induced biases have been observed in previous studies [16], [17], [18], [19], [20] and can hinder the sensitivity and specificity of CNV detection, since it has been associated with sequence repeats, proximity to chromosome ends [18], [19], [20] and GC content [18], [20]. In the procedure of WGA, GC content, in particular, can influence polymerase processivity and DNA priming so as to lead to false CNVs signal. This can be caused by over-amplification or under-amplification on GC-poor or GC-rich regions [18], [20]. Nowadays, massively parallel sequencing (MPS) has become an advanced approach for genomics research [21]. The power of whole genome sequencing (WGS) in profiling genome copy number landscapes makes it notably more advantageous over aCGH, as reported in previous studies [22], [23].

In this study, we developed a WGA-induced bias correction strategy based on GC bias description using two single cells isolated from the peripheral blood (PB) of YH, a healthy Chinese individual with a normal karyotype. We then established a practical bioinformatics pipeline, which detected CNVs at the single cell level through low coverage whole genome sequencing. GC correction to lessen WGA-induced bias, a binary segmentation algorithm for CNVs breakpoint location, and dynamic threshold determination for final CNV signals filtering constituted the core of this pipeline. Seven single cells isolated from PB or blastocysts with confirmed CNV results were recruited to examine the performance of this pipeline. Our method explores CNVs in single cells or limited DNA, which provides a practical approach for CNVs detection in the clinic.

## Results

### WGA-induced GC bias correction and pipeline establishment

**Figure 1:**
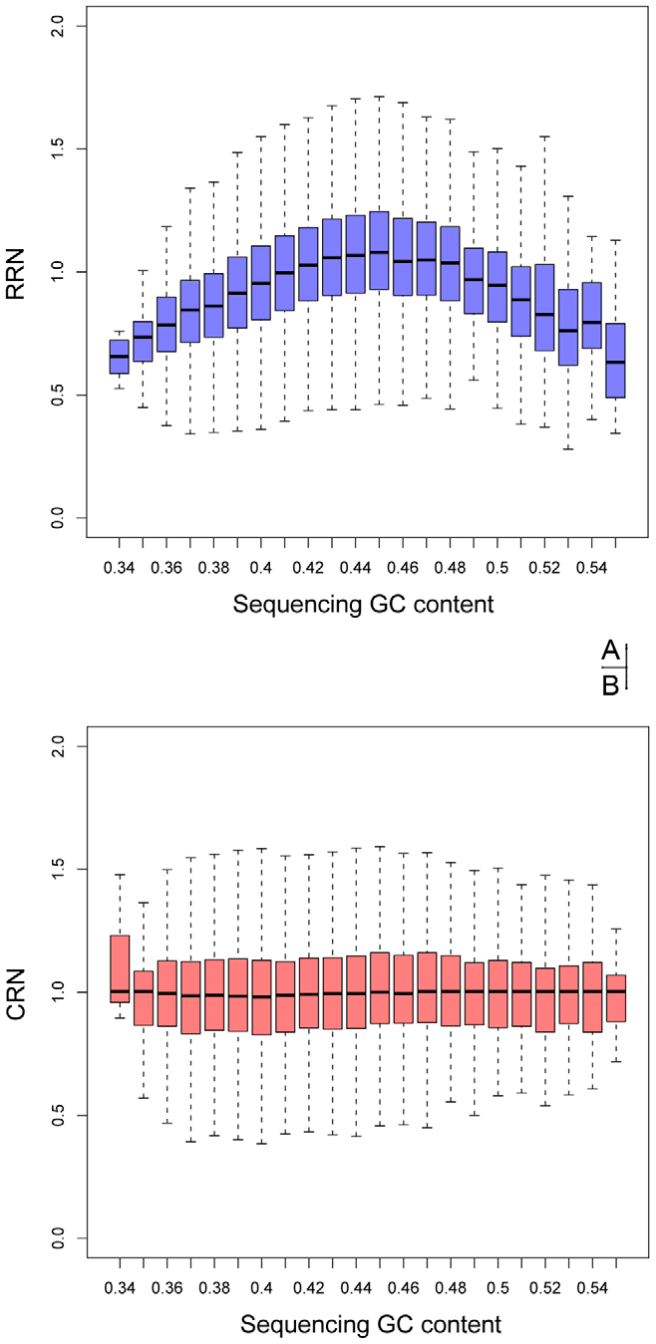
The distribution between RRN and Sequencing GC content before and after GC correction. The distribution of relative reads number (RRN, y-axis) and the corrected relative reads number (CRN, y-axis) were exposed as boxplot maps respectively with their sequencing GC content (x-axis).

In order to observe and correct GC biases in WGA, we isolated, amplified, and sequenced two single cells from the PB of YH, a healthy Chinese individual with normal karyotype [24], generating an average of 13.04 million single-end (SE) 50 bp reads of each cell (Table 1).We defined the quotient between the reads number of each observation window and the average reads number as relative read number (RRN), which ideally would be equal to one in diploid genome. By comparing the GC content and RRN, we discovered that the RRN tended to be less than average in genomic GC-poor (<40%) and GC-enriched (>48%) regions (Figure 1A), implying significant amplification bias of these regions.

**Table 1:** Sequencing data statistics.

Sample	GC content (%)	Reads number (M)	Map rate (%)	Coverage (%)	Depth (X)
SC 1	39.47	14.64	62.69	4.20	0.15
SC 2	40.74	14.64	80.66	7.60	0.19
SC 3	39.43	7.13	79.16	4.00	0.09
SC 4	41.53	9.65	84.80	6.60	0.13
SC 5	40.93	8.69	82.39	6.10	0.12
SC 6	43.40	16.69	74.44	9.50	0.20
SC 7	43.69	15.50	72.95	7.70	0.18
YH-1	41.75	11.75	78.13	6.80	0.15
YH-2	41.59	14.32	77.18	5.90	0.18

Based on this discovery, we developed a weighted correction strategy to remove the GC bias. RRN would be adjusted by a GC-related weighting coefficient, calculated using RRN in windows that shared same GC content (Methods and materials). After re-normalization, the corrected relative reads number (CRN) showed a much better uniformity of GC content, indicating a high efficacy of the correction (Figure 1B). Also, the self-defined GC-bias index proved that more than 99.90% of the GC biases were removed by our strategy. (Materials and Methods, Table S1)

**Figure 2:**
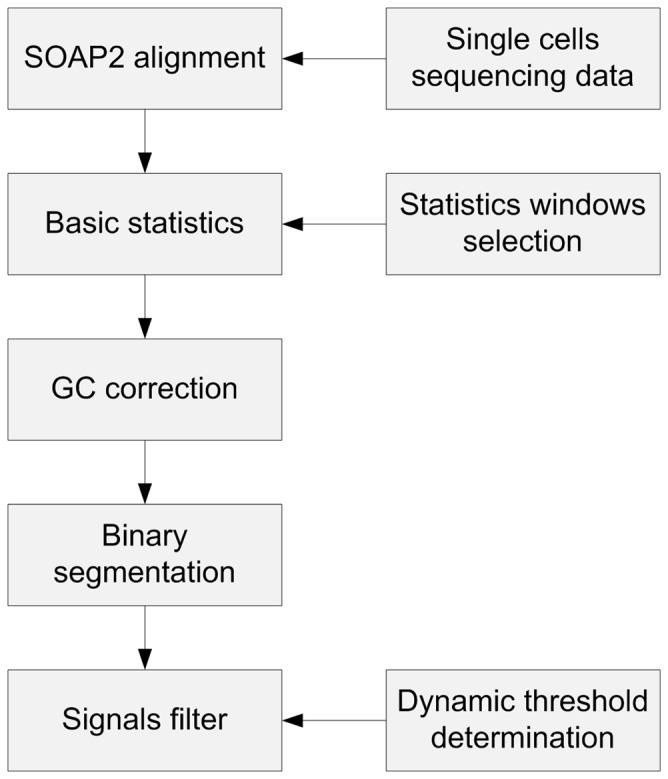
The comprehensive pipeline. This figure shows the structure of the method in this study for CNVs identification, composed of a sequencing reads alignment, GC bias correction, a binary segmentation algorithm and dynamic threshold determination for signals filtering.

After GC bias correction, we employed a binary segmentation algorithm to access higher accuracy of the localization of the CNVs breakpoints on the chromosome. Candidate breakpoints were obtained after initialization and the adjacent windows were merged to localize the optimized breakpoints. Considering the influence of different GC content on CNVs detection, we developed a dynamic threshold determination algorithm for the final signal filtering, which would improve the general sensitivity and specificity of CNVs detection. Finally, we established a comprehensive bioinformatics pipeline (Figure 2), which included sequencing reads alignment, GC bias correction, a binary segmentation algorithm and dynamic threshold determination for signals filtering.

### Evaluation of sensitivity and specificity

To examine our method, we isolated seven single cells from PB and blastocysts whose karyotypes were already confirmed by whole genome sequencing analysis or SNP-array analysis, respectively. These karyotypes were normal, had CNVs, or had aneuploidies. An average of about 10.92 million SE 50 bp reads were obtained for each single-cell samples and mapped to the human reference genome (HG18, NCBI Build36) using SOAP2 [25] (Table 1). The coverage of those sequencing reads in the genome ranged from 4.0% to 9.5%. All these single cell samples had received CNV analysis using the standard pipeline described above (Figure 2), and were used to evaluate the sensitivity and specificity of the method.

**Figure 3:**
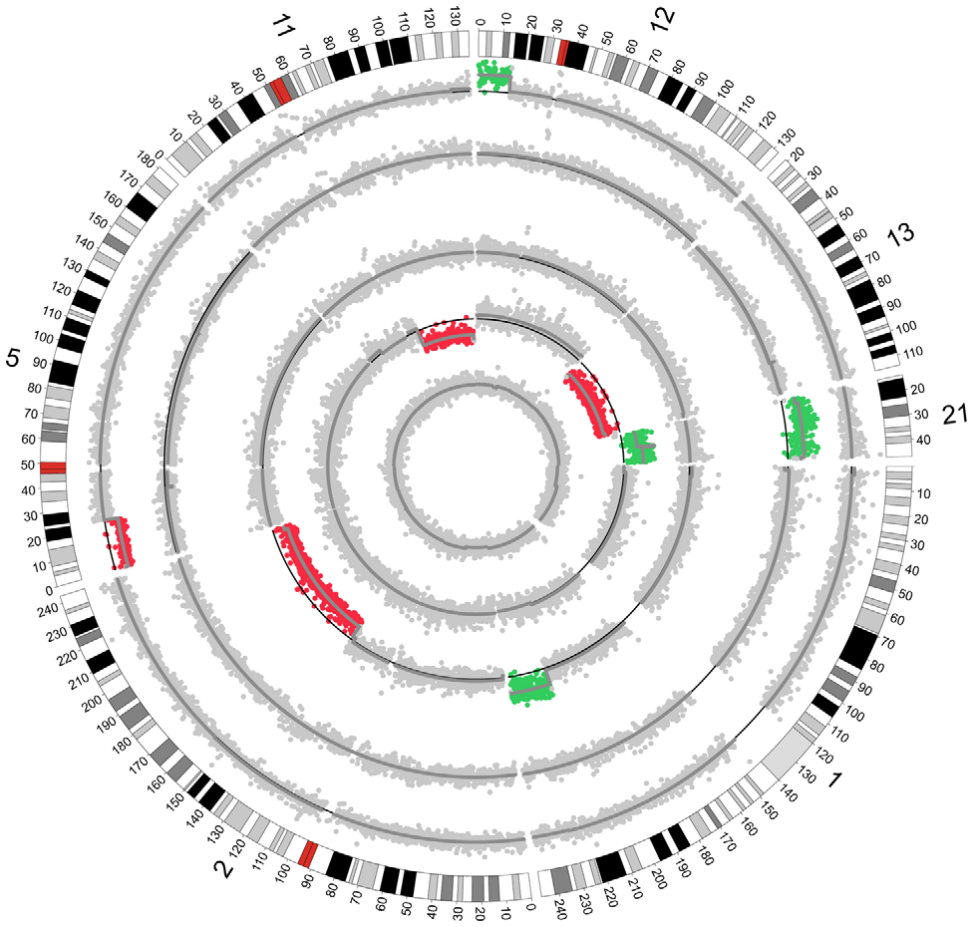
Performance of this method for test samples. This circular map shows the performance of our method on five single-cell samples with CNVs and aneuploidies. The outermost circle depicts the bands of chromosomes 1, 2, 5, 11, 12, 13, 21. The inner circles represent the samples SC1, SC2, SC3, SC4, and SC5. The color-coded dots represent the distribution of CRN, of which green and red show duplication and deletion, respectively. The dark grey lines show the CNVs after segmentation.

**Figure 4:**
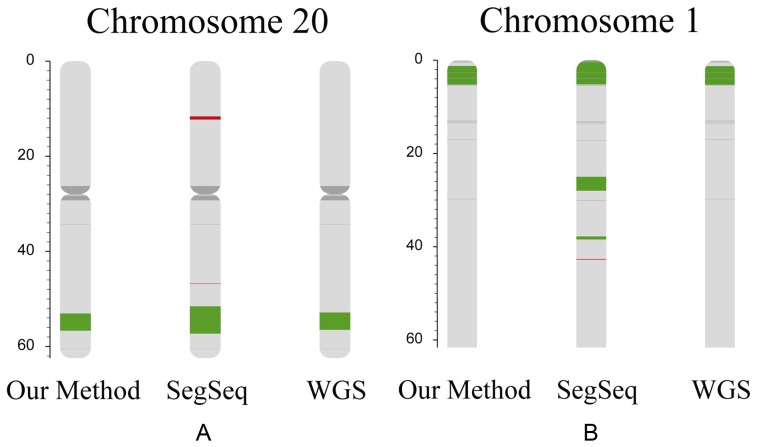
Comparison between this method and SegSeq for test samples. The figure below shows a comparison of our method's performance of CNVs identification and that of SegSeq's. Figure A shows the karyotype of chromosome 20 in single-cell sample SC 6 and figure B shows the karyotype of part of chromosome 1 in single-cell sample SC 7. The karyotypes were produced by our CNV identification method (left), SegSeq (middle), and WGS (right). The color red, green, dark gray and light gray represent deletion, duplication, N regions on the genome, and normal regions, respectively.

In total, six out of seven test single cell samples were identified with CNVs over 1 Mb or aneuploidies, including seven events of CNVs from five samples and three events of aneuploidies from two samples. Generally, the CNV results detected by our method were highly consistent with confirmed CNV results (Table 2). For the single-cell samples from PB or blastocysts, we successfully detected the CNVs and aneuploidies using our method (Figure 3 & Table 2). Especially, two smaller CNVs were also identified correctly in samples SC 6 and SC 7 (Figure 4 & Table 2). The two CNVs were a duplication of 3.94 Mb on chromosome 20 and a duplication of 5.47 Mb on chromosome 1. It indicates our method can identify CNVs (>1 Mb) correctly and the detection results can reach 99.63% sensitivity and 97.71% specificity (Table 3).

**Table 2:** Detected results of different methods for test single-cell samples.

sample	Our method	SegSeq	WGS/SNP-Array
SC 1	46, XY, del(5)(p14.2→pter), dup(12)(p13.1→pter)	46,XY, del(1)(p36.11→pter), del(5)(p14.3→pter), dup(12)(p13.1→pter), dup(13)(q21.1 q32.1), del(19)(p13)	46, XY, del(5)(p14.2→pter), dup(12)(p13.1→pter)
SC 2	47, XX, +21	47, XX, +21	47, XX, +21
SC 3	46, XY, dup(1)(q41→qter), del(2)(q21.1→qter)	46,XY, dup(1)(q41→qter), del(2)(q14.3→qter), del(19)(p13), del(19)(q13.2 q13.33)	46,XY, dup(1)(q41→qter), del(2)(q14.3→qter)
SC 4	46, XX, del(11)(q13.1 q25), −13, +21	46, XX, del(11)(q12.2→qter), dup(19)(p13), −13, +21	46, XX, del(11)(q13→qter), −13, +21
SC 5	46,XX	46,XX, dup(19)(p13)	46,XX
SC 6	46, XX, dup(20)(q13)	46, XX, dup(20)(q13), dup(19)(p13)	46, XX, dup(20)(q13)
SC 7	46, XX, dup(1)(p36.3)	46, XX, dup(1)(p36.3), dup(1)(p36.11)	46, XX, dup(1)(p36)

CNVs larger than 1 Mb in test samples were showed in this table.

**Table 3:** Comparison between our method and SegSeq.

Methods		Our method	SegSeq
The core of methods	Need a control (normal) sample	No	Yes
	Correction or Normalization	GC correction	Normalization based on control sample
	Segmentation algorithm	Yes	Yes
	Final signals filtering by specific method	Dynamic threshold determination for final signals filtering	No
CNV identification for test samples *	Sensitivity	99.63%	92.75%
	Specificity	97.71%	16.49%

* CNVs larger than 1 Mb in test samples were used to calculate the sensitivity and specificity.

**Figure 5:**
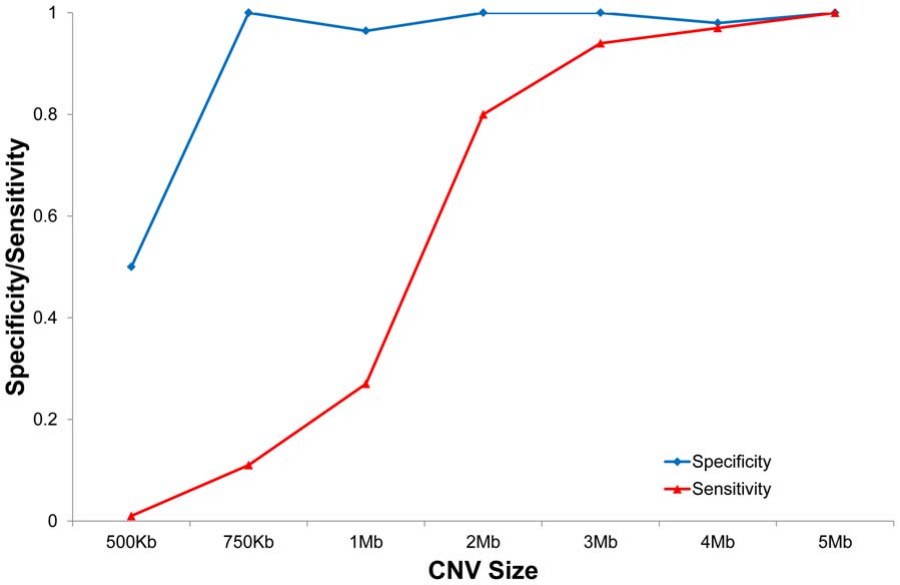
Evaluation of specificity and sensitivity for this method *in silico*. This figure shows the correlation between CNV size (x-axis) and specificity and sensitivity (y-axis) of this method. The color-code lines represent the sensitivity (red) and specificity (blue), respectively.

To further estimate the sensitivity and specificity of our method for CNVs detection on call level, we performed simulation *in silico* to depict the relationship between CNVs size and its performance (Materials and Methods). We first simulated CNVs, ranging from 500 kb to 5 Mb, on YH genome. Then, we applied our method to evaluate the sensitivity and specificity of our method. Overall, the sensitivity increased with the CNV size, and achieved about 94% for CNV over 3 Mb could be detected successfully (Figure 5). Also our method showed a high efficiency on decreasing the false positive rate, for example, the specificity of 750 kb CNV was as high as 95% (Figure 5). Additionally, we also studied our ability to map breakpoints accurately by this simulation. For this purpose, we calculated the minimum distance between the segmentation algorithm (using about 10M simulated reads) predicted breakpoint and the real breakpoint. The CNVs breakpoint precision analysis indicated that we could localize the CNV breakpoints within about 70 kb (median), which was associated with the size of observation windows.

### Comparison with comparative genomic strategy

We also compared the methodology and practical performance of our method with SegSeq [22], [26], a well-recognized CNVs detection method using short reads generated by massively parallel sequencing. Different strategies were conducted to reduce the significant influence of bias from WGA or sequencing procedure in both methods. We removed GC bias by a weighted strategy between observation (sequencing data) and expectation (the average). To contrast, SegSeq used a comparative genomic strategy to decrease the experimental variance between the case and control, which is similar to the principle of array-CGH. Another difference between two methods was the threshold determination for final signals filtering, which is dynamic in our method and fixed in SegSeq (Table 3).

We employed SegSeq to detect the CNVs in the seven test samples with suggested parameters (-W 400 –a 1000 –b 10), using the YH samples (YH-1 and YH-2) as control. A total of 16 CNVs events were identified in seven samples, and also three additional aneuploidies in two samples (Table 2). Almost all CNVs over 1 Mb were correctly detected in both methods, corresponding 99.63% and 92.75% of sensitivity for our method and SegSeq, respectively. However, the specificity for SegSeq was only 16.49% on CNVs larger than 1 Mb, which was far less than 97.71% of our method (Table 3). Most of the false positive signals of SegSeq were common in GC-poor chromosomes (such as chromosome 13) and GC-rich chromosomes (such as chromosome 19) (Figure S1), which simply could not be filtered by a fixed threshold. These signals could be explained by the significant GC bias in WGA described above. Moreover, in the case of single cells with smaller CNVs (Figure 4), our method showed a higher specificity for CNV detection than SegSeq. For example, on chromosome 1 of SC 7, a duplication of about 3 Mb on the cytogenetic band of p36 (24,736,617–27,719,432) was detected by SegSeq, but verified as a false signal (Figure 4B).

In brief, our method based on a weighted strategy had more efficient than the comparative genomics strategy to analyze CNVs using WGA data, only for detection of CNVs over 1 Mb.

## Discussion

In this study, we developed a practical bioinformatics method to detect CNVs at the single cell level through low coverage massively parallel sequencing. The method consists of GC correction, a binary segmentation algorithm and dynamic threshold determination. With our correction strategy, more than 99.90% of WGA-induced GC biases, which hinders single-cell CNVs analysis [18], [20], were removed (Table S1), indicating a high efficacy of GC correction. Finally, using the low coverage sequencing (4∼9.5%) data of test samples, our results were highly consistent with confirmed results with 99.63% sensitivity and 97.71% specificity (Table 3).

However, several improvements are still necessary for further studies. Firstly, our method shows a high false negative rate (FNR) on smaller CNVs (<1 Mb) (Figure 5). Also, the simulation results indicated that our sensitivity on smaller CNVs was strongly related to the number of observation windows (Figure S2). Using smaller observation windows could increase the sensitivity and improve the resolution of our method. In addition, false positive signal in smaller observation windows could be caused by the lack of data from low coverage of whole genome sequencing. Therefore, it is important for appropriate observation windows selection according to data production and the sensitivity and resolution of our method can also be improved by increasing the sequencing reads or target sequencing [27] using smaller observation windows. Secondly, except GC content, several factors, including chromosomal structure, repeat regions [18], [19], [20] etc., can also induce WGA-bias. Ideally, these biases would mostly relate to specific regions in human genome. Therefore, a population-scale normalization strategy, comparing the same observation window with data generated from the same batch, will be powerful for WGA-induced bias removal.

Our method can be utilized for research on somatic CNVs to explore cell evolution in cancers. Furthermore, it provides a potential solution for CNVs detection in clinical applications of single cell technology, such as PGD/PGS, using fetal nucleated red blood cell for non-invasive prenatal diagnosis [12], [13], or using PB for identifying circulating tumor cells for fast and convenient noninvasive cancer screening [28]. To meet these clinical needs, more samples are necessary to improve the sensitivity and specificity of our method. In conclusion, our method for CNVs detection using WGA and MPS data highlights that CNVs research at the single cell level and explores a potential solution for single cell applications in clinic.

## Materials and Methods

### Overall design and concept

To develop an effective bioinformatics strategy for CNVs detection at the single cell level through massively parallel sequencing, we generated sequencing data using single cells isolated from peripheral blood (PB) or blastocysts. The single cells received a standard degenerate oligonucleotide primer PCR (DOP-PCR) and Illumina sequencing at BGI-Shenzhen. After a short read alignment, we first tried to describe the WGA-induced bias. Considering the influence of the unique differences in the human reference genome sequence, windows selection was performed and well-selected windows were employed as the minimum observation unit. We described the WGA-induced bias using a relative reads number (RRN) and a newly defined statistic, the GC-bias index (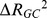). Based on this significant discovery, we developed a weighted correction strategy to lessen the influence of WGA-induced bias. Finally, a bioinformatics pipeline, which included GC correction for WGA-induced bias removal, a binary segmentation algorithm for CNV breakpoints identification, and dynamic threshold determination for a final signal filtering, was established to access more accurate CNVs.

### Sample recruitment, sample processing and single cell isolation

A total of nine single cell samples from eight individuals were collected at BGI-Shenzhen, CITIC Xiangya Reproductive & Genetic Hospital, and Nanjing Maternal and Child Health Hospital, including two from PB of YH with normal karyotype, one from PB of an individual with Down's syndrome, one from PB of an individual with Cri-du-Chat (CDC) Syndrome, two from PB of an individual with 1p duplication and 20q duplication, and three trophectoderm cell samples from blastocysts. All those samples were karyotyped by G-banding or FISH in local hospitals before this study.

Each participant was recruited with informed written consent and approval of the Institutional Review Board of BGI-Shenzhen, for those trophectoderm samples the parents were also provided informed consent under the protocol approved by the Ethics Committee of the CITIC Xiangya Reproductive & Genetic Hospital. For three of the participants less than five years old, informed written consent was obtained from their guardians. All the potential participants or their guardian were provided with the detailed information of this study, including the benefits and risks of our technology development in written information consent form, and also were informed of the rights to privacy. The results of this study would not feedback to the participants or their guardian; therefore all potential participants or their guardian who declined to participate or otherwise did not participate were eligible for treatment and were not disadvantaged in any other way by not participating in this study.

5–10 mL of PB was collected in EDTA-coated tubes at BGI. 10 ul blood samples for single cell isolation were frozen in −20°C. The blood was thawed and washed with 500 ul of PBS with centrifuged at 1,000 rpm for 10 minutes at room temperature. The supernatant was discarded, and the nucleated cells were re-suspended in PBS.

PBS with 5% BSA was used for droplet preparation in culture dishes. We used mouth-controlled pipettes to transfer and dilute the nucleated cells suspension in droplets under a microscope, and isolated a single nucleated cell into 200 ul tubes containing 1.5 ul of alkaline lysis buffer. The tubes with single cells were frozen in −80°C until further processing.

For blastocyst biopsies, the embryos expanded trophectoderm (TE) protruding through the opened zona on day 5 or 6 were chosen. Three to eight herniating TE cells were aspirated into the lumen of a pipette (internal diameter: 30 µm) and detached from the blastocyst by firing a laser at the area of constriction. They were then washed several times in wash buffer and preserved in 200 ul PCR tubes.

### Data generation and basic process

The single cells were amplified with the GenomePlex Single Cell Whole Genome Amplification Kit (Sigma Aldrich) according to the manufacturer's instructions. The cell WGA was quality controlled by PCR with primers for housekeeping genes including PRDX6, RPL37a, ADD1, PSMD7, and ATP5O.

In total, two YH cells (YH-1 & YH-2), one T21 cell (SC 1), one CDC cell (SC 2), two micro-duplication cells (SC 6 & SC 7) and three single-cells from blastocysts (SC 3, SC 4, SC 5) were qualified for further process in this study. After WGA, the products were used to prepare a library of 350 bp insert size, and received whole genome sequencing in Hiseq2000 platform with single-end (SE) 50 bp. All the raw sequencing data had submitted to NCBI SRA (http://www.ncbi.nlm.nih.gov/sra) and the Submission ID is SRA060638.

In the basic data process, reads after WGA-adapter removal were mapped to the reference human genome (Hg18, Build36) using SOAP2 [25] with maximum two mismatches [−v 2]. Afterwards, we removed low quality alignments, such as PCR duplications (i.e. the identical reads) and non-unique alignments.

### SNP array and Whole genome sequencing (WGS) analysis

To evaluate the performance of our method, we used two different methods as gold standard, SNP array for single-cell samples and WGS for peripheral blood (PB) samples.

Van et al. reported the SNP array analysis can identify CNVs as small as 150 kb (>5 SNPs) for single cell [29]. Therefore, it is an appropriate gold standard to compare the accuracy of detecting CNV larger than 1-Mb in this study. For three single-cell samples (sample SC3 to SC5), SNP array analysis was conducted according to the manufacturer's instructions. Each DNA sample was hybridized to the Gene Chip Mapping Nsp I 262K microarray (Affymetrix Inc., Santa Clara, CA). Approximately 260,000 SNP signal intensities for each test samples were compared computationally with averaged signals from 30 previously evaluated normal female reference samples. Copy number analysis was performed by Gene Chip Genotyping Analysis Software (GTYPE) with default parameters to minimize the noise hybrid signals.

Derek et al. showed that WGS method can achieve a sensitivity of approximately 100% when detected CNVs larger than 200-kb using ∼10M reads [22]. Thus, we employed WGS methods as gold standards for PB samples to detect CNV larger than 1-Mb. The rest six PB samples, including YH and the individuals with Down's syndrome, Cri-du-chat syndrome and two individuals with micro duplication syndrome, were used for DNA extraction and whole genome sequencing analysis with 350 bp insert size library and single-end 50 bp sequencing in Illumina HiSeq2000 platform, generating average 10M reads for each sample. The sequencing data was mapped into the reference genome (Hg18, Build 36.3) and non-unique mapped reads and PCR duplication were removed. Then, we recruited these two YH samples as controls to perform copy number analysis for the other four samples by SegSeq (parameters: -W 400 –a 1000 –b 10) [22], [26].

### Windows selection

To lessen uncertain factors caused by data volume and characteristics of the human reference genome, such as GC content and uniqueness, we performed an optimized dynamic observation windows selection. First, the average size of the windows was determined by considering the GC content that is characteristic of the human reference genome and our low coverage sequencing strategy. Since it had been reported that long DNA segments (>300 kb) have relatively homogeneous GC content [30], the average size should be less than 300 kb to access vivid GC content distribution for observation or correction. However, the low coverage sequencing strategy (about 10 million reads per sample) would lay more uncertain influence in smaller genomic regions. Therefore, we decided to divide the human genome into non-overlapping observation windows with an average size of about 150 kb. Second, after considering uniqueness, we performed a simulation to obtain the dynamic observation windows. We divided the reference genome (HG18, Build 36) into sliding SE50 simulated reads and mapped them to the genome with a maximum of two mismatches and reserved unique mapped reads. When constructing these windows, we allowed the windows to share the same simulated reads numbers instead of the same window size to achieve higher comparability of suitable expected sequencing reads number among windows. Finally, we got 18,743 observation windows, each sharing 140,000 simulated reads (Figure S3).

### WGA GC bias description and correction

To describe the GC bias in WGA, we defined several statistics, including the average sequencing GC content (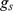), average reference GC content (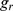) and unique mapped reads (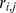) in each window. Subscript 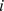 and 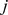 represent the different windows and samples, respectively. The windows with no reads and zero GC percent are ignored. Let 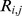 represents the relative reads number (RRN), which is calculated by 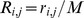, where 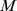 is the global average number of sequencing reads in each window on autosomes. The effect of GC bias on the RRN was defined as 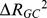, which implied the average deviation between the observed RRN to its expected, where 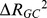 is calculated by the following formula: 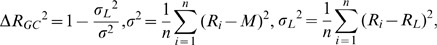
and 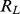 represents the optimal prediction, which was obtained via a loess regression fit of the relative reads number (RRN) against the GC content, rounded to the nearest 0.5% increment. [22].

Based on this discovery, we developed a weighted correction strategy in the following way. The sequencing reads within the window 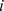 with GC content (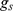 and 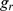) were assigned with a weight 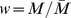, where 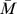 is the average number of sequencing reads in each window which is calculated for every 1% GC content in sequencing data (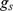) and every 1% GC content in reference genome (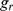). Then, the corrected reads number (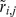) in each window was achieved with the following formula, 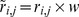. Afterwards, we defined the correction relative reads number (CRN, 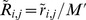, where 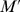 is the global average CRN) as the normalized statistics for the following analysis.

### Binary segmentation algorithm for CNVs breakpoints identification

After removing the WGA-induced bias, we developed a binary segmentation algorithm to detect CNVs breakpoints with low coverage massively parallel sequencing. The binary segmentation algorithm consisted of initialization and iterative merging between adjacent segments.

#### Initialization

We calculated the significance of differences between the two sides of each window with a *run-test*. Moreover, 100 CRNs were employed in the left and right side of each window for difference significance statistics and each window was assigned a *p*-value. Then, the 3,000 windows with minimal *p*-value were selected as ordered initialized candidate breakpoints (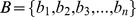).

#### Iterative merging between adjacent segments

Each breakpoint 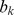 was associated with a left segment from 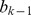 to 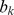, and a right segment from 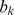 to 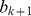 [22]. We estimated the difference between the left and right segment of each window with a *run-test*, where the *p*-value was denoted as 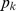. The candidate breakpoint with the most insignificant difference (the largest *p*-value) would be removed from 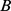, indicating that segments of the two sides of this breakpoint were merged. The iteration calculation was performed until the *p*-value of each breakpoint was less than the final *p*-value cutoff (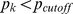), where we choose the final *p*-value cutoff (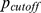) from a control by the methods described below.

### Dynamic threshold determination for final signal filtering

To minimize the false signals and misdiagnosis of CNVs, we defined a cutoff threshold for the average CRN between two breakpoints after segmentation. Since the regions with same sequencing GC content had similar variation trends in WGA, based on central-limit theorem, we calculated the lower and upper quantile (alpha = 0.05) of CRNs with the same sequencing GC content as the deletion and duplication cutoff threshold respectively.

### Evaluation of sensitivity and specificity for test samples

To assess the efficacy of this method for CNVs detection, we calculated the sensitivity and specificity through seven test samples in the following way:
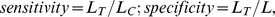
where 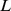 represents the total length of CNVs detected by this method, 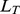 represents the length of true CNVs detected by this method, and 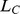 represents the length of CNVs confirmed by SNP-array or whole genome sequencing analysis.

### Simulations and breakpoint precision analysis *in silico*

To get the overall sensitivity and specificity of our method on call level, we performed a simulation on YH genome *in silico*. *(I) Candidate genomics region selection.* We first filtered the N regions on YH genome. Then we randomly selected regions as candidate regions for further CNV simulation. To mimic the influence of YH “true CNVs” in further simulation, we analyzed these candidate regions using WGS data of YH PB with SegSeq and excluded those regions overlapped with “true CNVs” on YH genome. The remained candidate regions would be used to CNVs simulation. *(II) CNV simulation.* We then randomly simulated CNVs ranging from 500 kb to 5 Mb based the candidate regions above on YH-1 and YH-2. We simulated totally 100 CNVs for each size. Under the consideration of sequence volume, we extract 10M sequence reads from YH-1 and YH-2 respectively. RRNs on selected regions would multiply by 0.5 (deletion) or 1.5 (duplication) directly. Standard GC correction, binary segmentation and dynamic threshold determination were performed to detect these simulated CNVs. *(III) Sensitivity/specificity statistics and breakpoint precision analysis.* The sensitivity and specificity were calculated by the following formula:
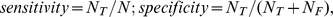
where 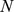, 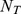, 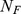 represent the number of simulated CNVs (100), true signals of CNVs and false signals of CNVs, respectively. Also, we calculated the minimum distance between the predicted and simulated CNV breakpoints to reveal the breakpoint precision of our method.

## Supporting Information

Figure S1 **Comparison between this method and SegSeq for test samples.** The figure below shows a comparison of our method's performance of CNVs identification and that of SegSeq's and the distribution of RRNs and CRNs against GC content. The part A & B show the karyotype of part of chromosome 5 and 19 and the part C & D show the distribution of RRNs and CRNs against GC content in single-cell sample SC 1. The part E & F show the karyotype of part of chromosome 1 and 13 and the part G & H show the distribution of RRNs and CRNs against GC content in single-cell sample SC 3. The karyotypes were produced by our CNV identification method (left), SegSeq (right). The color red, green, dark gray and light gray represent deletion, duplication, N regions on the genome, and normal regions, respectively. The black dots and green triangles on right figures show the distribution of RRNs and CRNs against GC content on genome and false positive region (on the chromosome 19 of SC 1 and the chromosome 13 of SC 3).
(TIF)

Figure S2 **Evaluation of specificity and sensitivity for this method by simulation.** This figure shows the correlation between CNV size (x-axis), the number of observation windows (color-code lines) and performance (y-axis) of this method.
(TIF)

Figure S3 **The distribution of observation window size.** This histogram shows the distribution of observation window size using in this study. The x-axis and y-axis represent the window size (bp) and frequency of the same window size, respectively.
(TIF)

Table S1 Effect of GC bias on the relative reads number (RRN).
(DOC)
