# Supplementary figures and images for: A Single Cell Level Based Method for Copy Number Variation Analysis by Low Coverage Massively Parallel Sequencing
Source: PLoS One. 2013 Jan 23;8(1):e54236. doi: 10.1371/journal.pone.0054236 (PMC3553135; doi:10.1371/journal.pone.0054236)

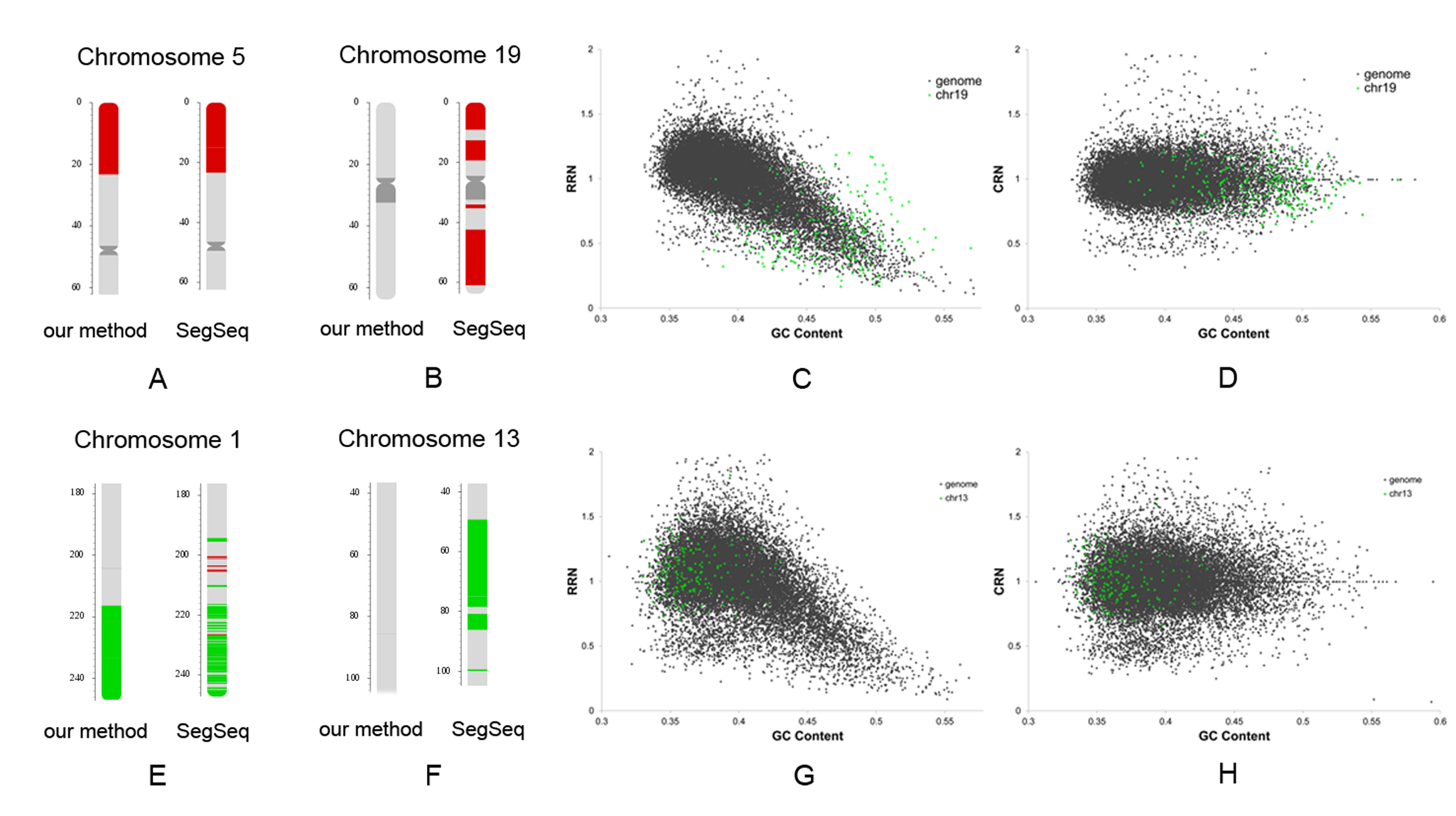

Supplement: Figure S1 — Comparison between this method and SegSeq for test samples. The figure below shows a comparison of our method's performance of CNVs identification and that of SegSeq's and the distribution of RRNs and CRNs against GC content. The part A & B show the karyotype of part of chromosome 5 and 19 and the part C & D show the distribution of RRNs and CRNs against GC content in single-cell sample SC 1. The part E & F show the karyotype of part of chromosome 1 and 13 and the part G & H show the distribution of RRNs and CRNs against GC content in single-cell sample SC 3. The karyotypes were produced by our CNV identification method (left), SegSeq (right). The color red, green, dark gray and light gray represent deletion, duplication, N regions on the genome, and normal regions, respectively. The black dots and green triangles on right figures show the distribution of RRNs and CRNs against GC content on genome and false positive region (on the chromosome 19 of SC 1 and the chromosome 13 of SC 3). (TIF) [file pone.0054236.s001.tif]

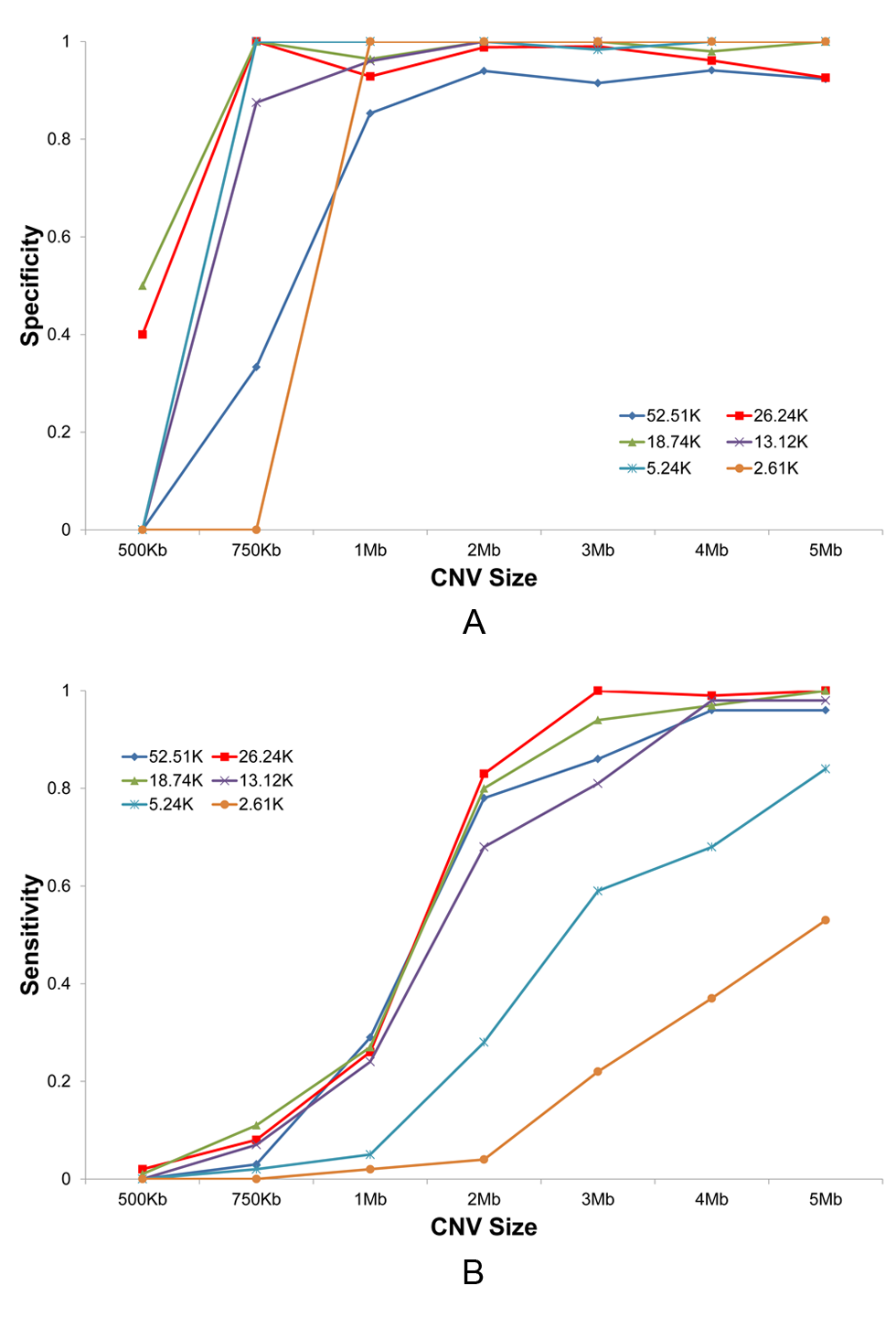

Supplement: Figure S2 — Evaluation of specificity and sensitivity for this method by simulation. This figure shows the correlation between CNV size (x-axis), the number of observation windows (color-code lines) and performance (y-axis) of this method. (TIF) [file pone.0054236.s002.tif]

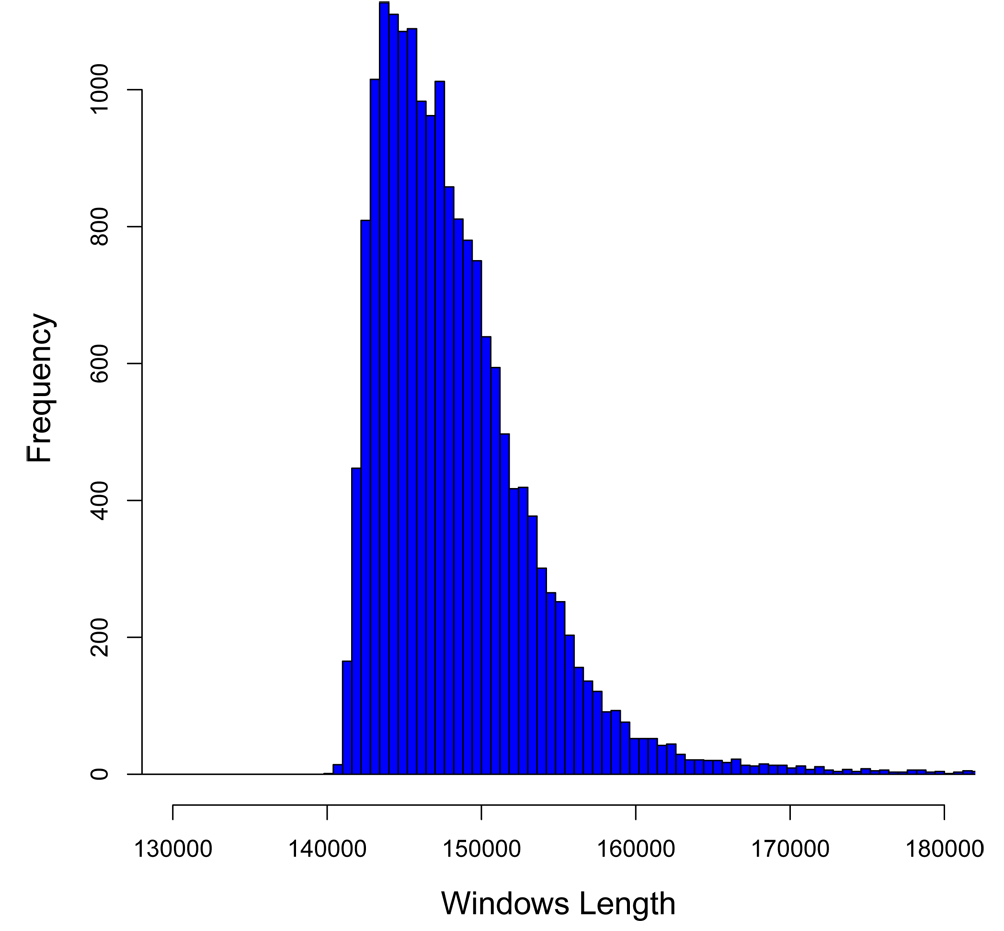

Supplement: Figure S3 — The distribution of observation window size. This histogram shows the distribution of observation window size using in this study. The x-axis and y-axis represent the window size (bp) and frequency of the same window size, respectively. (TIF) [file pone.0054236.s003.tif]
